# Determination of Motivating Factors of Urban Forest Visitors through Latent Dirichlet Allocation Topic Modeling

**DOI:** 10.3390/ijerph18189649

**Published:** 2021-09-13

**Authors:** Doo-San Kim, Byeong-Cheol Lee, Kwang-Hi Park

**Affiliations:** 1 Graduate School of Tourism, Event, and Convention Management, Kyonggi University, Seoul 03746, Korea; spero410@gmail.com (D.-S.K.); 2bclee@kgu.ac.kr (B.-C.L.); 2 Department of Nursing, Gachon University, Incheon 21936, Korea

**Keywords:** urban forest, motivation, LDA, topic modeling, scale development

## Abstract

Despite the unique characteristics of urban forests, the motivating factors of urban forest visitors have not been clearly differentiated from other types of the forest resource. This study aims to identify the motivating factors of urban forest visitors, using latent Dirichlet allocation (LDA) topic modeling based on social big data. A total of 57,449 cases of social text data from social blogs containing the keyword “urban forest” were collected from Naver and Daum, the major search engines in South Korea. Then, 17,229 cases were excluded using morpheme analysis and stop word elimination; 40,110 cases were analyzed to identify the motivating factors of urban forest visitors through LDA topic modeling. Seven motivating factors—“Cafe-related Walk”, “Healing Trip”, “Daily Leisure”, “Family Trip”, “Wonderful View”, “Clean Space”, and “Exhibition and Photography”—were extracted; each contained five keywords. This study elucidates the role of forests as a place for healing, leisure, and daily exercise. The results suggest that efforts should be made toward developing various programs regarding the basic functionality of urban forests as a natural resource and a unique place to support a diversity of leisure and cultural activities.

## 1. Introduction

According to the UN DESA [1], 55% of the global population resides in urban areas. Individuals are increasingly migrating from rural to urban areas, and 68% of the population is predicted to reside in urban areas by 2050. The report also pointed out the importance of an integrated policy regarding the different problems of housing, transportation, education, and healthcare in line with gradual urbanization. In the U.S., the importance of “urban forest” was recognized, and in 1978, the “Cooperative Forestry Assistance (CFA) Act” was enacted for legislating various support and forestry management programs [2].

Although there has not been a firmly agreed definition of urban forests, it is commonly agreed that urban forests differ from forests outside urban areas in terms of their existence in dense areas of human settlements such as large cities and towns [3]. This study defines urban forests as trees, forests, green space, and associated ecosystem and cultural components growing in and around cities as well as communities where people live, work, and play.

The recent rise in concerns for various diseases and pollution along with the COVID-19 pandemic have enhanced healthcare interests in South Korea [4]. Compared to the rapid urbanization and the emergence of cities with relatively high population density, the number of forests within the living districts has not increased significantly. Thus, to provide pleasant living environments and places to rest, a comprehensive law for creating and managing urban forests was enacted in Korea on 9 June 2020, as part of various efforts to ensure a diversity of benefits of forest resources for urban citizens in the vicinity [5].

The specific factors that motivate urban forest visits are being prioritized to highlight the functions and benefits of urban forests and to develop suitable programs for visitors. For these reasons, studies have investigated forest-related policies, the positive effects of forests on health, and the economic impact of forests. However, there is a lack of independent studies focusing only on the motivation of visitors to urban forests. In fact, because the actual urban forest visitors prefer short-distance travel to places in close proximity to city centers or urban living districts, this type of visit needs to be distinguished from the visit to conventional forests such as healing forests, natural recreation forests, and national parks. Most studies on forest resources investigated the visits to the aforementioned conventional forests (healing forests, natural recreation forests, national parks, etc.) rather than the urban forests themselves [6,7,8].

Previous studies identifying the factors that motivate forest visitors have generally used a conventional scale development approach that relies heavily on the literature review of motivations, the survey of visitors, and in-depth interviews [9,10]. However, there are several limitations to the conventional survey-based approach to extract the motivating factors: First, it is difficult to ensure an adequate sample size due to sampling limitations such as time and cost constraints; second, it is difficult to generalize the results of studies that consider diverse types of urban forests, as the urban forest types vary according to the city type and as the social and physical aspects of urban forests vary greatly [11] and third, it is difficult to ensure the representability of the motivating factors at different time periods for each urban forest, as a survey-based study is mostly dependent on a cross-sectional study design.

Thus, this study aimed to identify the factors leading to the visit to urban forests through a novel method based on social big data that allows a large sample size, including the visitors’ experience of different urban forest types and the representability of the temporal range. Specifically, the text data of social media containing the keyword “urban forest” were extracted as big data, and latent Dirichlet allocation (LDA) topic modeling was applied to draw each specific topic (theme) to identify the factors that motivate urban forest visitors. Since city residents are likely to experience the urban forests on a more regular basis than other natural forests outside urban areas [3], it is expected that their motivations may vary. Therefore, identifying and understanding the motivation factors of urban forests visitors can provide insights on designing and managing functions and benefits of urban greenspace based on the needs of visitors.

## 2. Literature Review

### 2.1. Urban Forests

Urban forests provide leisure services such as recreation and physical and psychological recovery to the urban residents who are prone to various psychological and emotional problems such as stress; they also function as ecosystems. Therefore, the importance of urban forests has been emphasized as a means to solve the different problems induced by the complexity of urban living [1].

An urban green space (UGS) functions as a critical resource that alleviates the negative psychological and emotional effects of urban life [11,12,13]. Planting trees in an urban area has esthetic and recreational values, while the resulting area may be managed as a wildlife habitat. It may also reduce the burden on natural forests and enhance the quality of life of urban residents [12].

With the recognition of the importance of UGS, efforts have been made to provide a diversity of benefits related to urban forests to urban residents in South Korea. In June 2020, urban forests were defined as “forests and trees produced and managed to enhance the health and recreation as well as emotional cultivation and field experience of people in an urban area”, through the enactment of the Regulation of the Production and Management of Urban Forests, etc., while various policies are underway towards the use of urban forests as a place for refreshing environment, rest, and recreation, as well as health improvement and emotional cultivation. Furthermore, several projects for urban forest production focus on preventing the diffusion of fine dust from the source throughout the living district and reducing the fine dust concentration, thereby facilitating the supply of clean air [14]. Various programs related to forests have also been applied to urban forests to improve the immunity and the physical and psychological health of the people [15].

As such, the interest in applying urban forests in leisure activities has increased, but so far, only a few independent studies have been conducted regarding the motivating factors of visiting urban forests that are uniquely distinguished from other forests as the resource closely related to urban residents, while many similar studies have reported on the conventional motivations behind forest resources such as green areas, forestry, healing forests, and national parks.

### 2.2. Motivations to Visit Urban Forests

The factors that motivate urban forest visitors were analyzed in previous studies from various perspectives, as they targeted visitors to urban forestry or UGS. Studies regarding the factors that motivate the visit based on the review of papers published in South Korea and overseas have mainly included those on urban forest, forest park, national park, and urban green space. The scope of previous studies on urban forests included urban forests, healing forests, natural recreation forests, green areas, and national parks. Table 1 summarizes the main motivating factors for urban forest visitors. 

Analyzing the previous studies showed that the identified factors in general could be accounted for by the push and pull dynamics based on tourism motivation, a motivation theory. The push factors indicate the internal factors of motivation, including escape from daily life, rest, respect, health, and adventure-related desires. The pull factors indicate the external factors of motivation including tourism resource, tourism facility, novelty, convenience, and reputation [23]. In many studies as listed in Table 1, health, exercise, rest, walking, healing, natural exploration, and escape from daily life were the main factors of motivation, with a high rate of revisit by local residents.

Visiting an urban forest may satiate the need for privacy among urban dwellers [16]. Pan and Ryan [9] classified the following five dimensions of motivation factors: rest, social interaction, sense of belonging, technical prowess, and knowledge, based on the study of visitors to the Pirongia Forest Park in New Zealand using the leisure motivation scale. Of note was the high rate of revisits by local residents. In the study by Liu, Li, Xu, and Han [17], the motivating factors of urban forest visitors in Beijing, China, were physical exercise, rest and mood change, interaction with nature, visiting with a child, enjoying good weather, visiting cultural heritage sites, clean and fresh air, reading books, meeting friends, and walking dogs. Among them, physical exercise (27.4%) and rest and mood change (26.7%) were the most common factors, and for accessibility, it was suggested that the park should be located within 1000 m of the visitors’ homes. Zhai, Baran, and Wu [18] broadly divided motivation into socially oriented and nature-oriented, where the main factors were playing with children, interaction with nature, rest, and meeting family and friends.

Most studies conducted in South Korea have focused on the motivation to visit healing forests. Nam and Lee’s [8] study on the visitors of healing forests suggest that the push factors were physical, psychological, and social motivations and the pull factors were natural environment, ancillary facilities, programs, and field experiences. Kim and Kim [7] identified six motivating factors: natural experience, calmness, natural exploration, health, social interaction, and emotional relaxation; among these, natural experiences exhibited the highest percentage, indicating the importance of the natural environment and healing forest-related programs and experiences.

The motivations related to natural recreation forests were categorized in a study on hiking tourists as part of nature-based tourism, as follows: enjoying the natural environment, escaping from daily routine, new trips, healthy life, and a sense of intimacy [19]. In a study by Choi and Lim [6], the correlation with visitor satisfaction was shown through the push factors, education of a child, escape from daily life, novelty, natural exploration, and social interaction, and the pull factors: use of programs or facilities, amenities, and ecology or environment.

Stigsdotter and Grahn [20] showed that the most preferred activity among the most stressed visitors of a UGS was rest, followed by animal-related activity and forest walk; these highlighted the importance of creating UGS to promote outdoor activities. In Jim and Shan [13], UGS visitors in Guangzhou, China, were motivated by factors directly related to personal or family benefits, such as enhanced health, child development, and reduced stress. 

Finally, Uysal, McDonald, and Martin [21] reported that the motivating factors for visiting national parks were rest or hobby, novelty (new activity), reinforced social relationships, escape from daily life, and reputation, while Confer, Vogelsong, Graefe, and Solan [22] categorized the factors into studying nature, relieving stress or solitude, pursuing a challenge, adventure, or sense of accomplishment, self-perception, career position, sense of belonging, fun, and autonomy. The national park visitors were shown to pursue not only the benefits of rest and stress relief but also the new experience of a diversity of lifestyles based on originality, especially for those who are yet to visit a national park.

As discussed before, the motivating factors of visiting forest resources could not be clearly differentiated despite the unique characteristics of each forest resource. The reason may be the use of the conventional scale-development method, where the items are first extracted from previous studies on forest resources to identify the motivating factors; then, the final factors are determined through a survey of actual visitors. In other words, since most motivation-related studies rely on the broad frame of motivating factors that already secured validity and reliability, it would have been difficult to derive differentiated factors. Moreover, the factors analyzed based on a survey present a challenge of adequate sample size to reflect the diverse motivating factors of visiting forest resources. Another limitation comes from the study being based on a cross-sectional design, as the survey is performed at a specific time, preventing the variation in motivating factors at each different time of visit to be reflected.

For these reasons, this study used social data containing the experience of visiting an urban forest obtained from social media (blogs) that guarantee adequate samples, coverage of the wide range of time, and minimization of subjective judgment for extracting the motivating factors of visiting urban forests.

### 2.3. Usefulness of Big Data

The decision to visit a specific destination is influenced by experiential data in social media, with social media reviews becoming increasingly important. Accordingly, research interest in understanding visitors’ various behaviors based on social media data has continuously increased [24,25]. Hence, it is now essential to use social media data to analyze the behaviors of visitors, such as that factors potentially attractive to visitors, reasons driving the visitors to specific destinations, and their experiences after the visit. Notably, in the case of visitors to forest resources, the information regarding the potential target destination is researched on the website to plan the visit before deciding the destination, while after the visit, the experience is recorded on the website. For blogs, basic information regarding a particular destination is provided, while the motivation for visiting the place, the surrounding scenery and facilities, and the experiences are broadly stated. Hence, the analysis of such data is useful for studying the motivating factors of visitors to urban forests (e.g., [15,26,27,28]).

However, as previously described, studies regarding the motivation to visit urban forests have mainly used conventional methods (e.g., survey, 1:1 in-depth interview, etc.) with consequent limitations such as the difficulty in analyzing the variations over time due to the samples collected for a set period and the errors in generalization due to inadequate sample size. The use of social media data could be an alternative to overcome a number of limitations related to data collection that frequently occur when applying conventional methods [29]. Furthermore, as it is possible to collect a large body of accumulated data on the experience of visiting urban forests over time, time-dependent variations can also be incorporated into the analysis.

## 3. Methodology

### 3.1. Site Selection and Sampling (Web Crawling)

To identify the motivating factors of urban forest visitors, big data containing visitors’ experiences were collected. Blog is an important medium that relays information regarding the topic of interest easily and rapidly, while opinions can be shared and goods or services can be selected. Thus, two websites with accumulated data on visitors’ experiences, Naver and Daum, with 60% share of portal sites in South Korea based on 2020, were selected, and the entire text data from the blogs that contain the keyword “urban forest” were collected for the period between 1 July 2019 and 30 June 2020. The 38,056 cases of the urban forest blog data from the most recent year and 19,393 cases of the data of 11 forests selected and promoted by the Korea Forest Service were collected, totaling 57,449 cases of data samples.

The method of data collection was the Python-based Web Crawler program run independently in this study, and the “Web Title” and “Meta Tag Description” of the blogs searched based on the keyword “urban forest” were collected.

### 3.2. Data Pre-Processing (Text Mining)

After the cleaning the first collected set of 57,449 cases based on the procedures of big data analysis, the final 40,110 cases were used in the analysis. Without appropriate pre-processing, the collected data may pose problems of incomplete data that lack a necessary attribute, noisy data that show an abnormal value outside the set scope, and inconsistent data that may arise in compiling several datasets [30]. The goal of the cleaning is to prevent the subjective judgment of the investigator as much as possible and to obtain the results with the highest accuracy in line with the study purpose. The pre-processing involved the following steps to remove the data not in line with the study purpose and to convert the informal data into an analyzable format.

#### 3.2.1. Noisy and Incomplete Data Cleaning

In the first cleaning process, texts that did not agree with the study purpose, such as advertisements (e.g., real-estate data), were eliminated. Texts related to real estate, rent, studio, sales, lease, or monthly rent were excluded based on the related regional posts and keywords, as they may add other forms of meanings than the intended ones if left alongside the texts related to the keyword “urban forest”. In this way, the data were cleaned so that only the topic (factor) related to “urban forest” with an intact meaning could be drawn.

A blogger may write the contents related to the actual experience and review as well as marketing contents for commercial purposes, hence the keywords, despite containing “urban forest”, could lead to an unrelated, commercial blog. Therefore, it is crucial to perform data cleaning prior to the analysis.

#### 3.2.2. Morpheme Analysis and Stop Word Elimination

Prior to analysis, the MeCab was used to construct the “User Dictionary”, whereby the process of structural conversion of the informal data was performed. Following the conversion to the document-term matrix, the open api KoNLPy was used for morpheme analysis, a process of segmentation of phrases or sentences into the smallest semantic unit of morphemes, including both the lexical and syntactic senses. The term “morpheme” means the “first semantic unit”.

The process of “User Dictionary—Morpheme Analysis—Stop Word Elimination” is crucial for extracting accurate results in line with the study purpose, and during the process, care should be taken to prevent data loss. Each separated word was screened for common nouns, proper nouns, bound nouns, counter nouns, numerals, and pronouns, while adjectives (e.g., food, bad, easy, difficult, etc.) and post-positions as well as special letters, emoticons, symbols, and foreign words are eliminated to leave the pure Korean morphemes.

The process involves stop word elimination, as stop words do not confer significant contextual meanings despite being essential components of sentences in the processing of natural language. The names of specific brands or regions (e.g., Seoul, Gyeonggi, Gangwon, Busan, Jeju, etc.) that show no association with the study purpose, despite the high frequency of detection, were eliminated. Words unrelated to the factors of urban forest visitors (e.g., renewal, development, architecture, construction, etc.) were repeatedly extracted to be eliminated.

#### 3.2.3. LDA Topic Modeling

**Figure 1:**
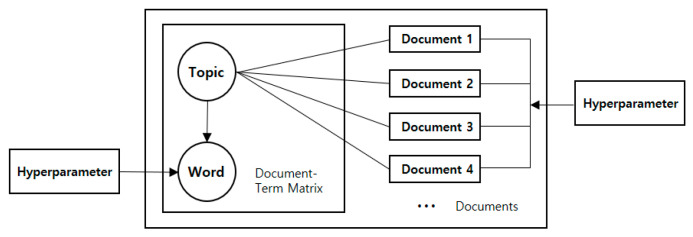
LDA topic generation.

The clean text data was applied to the LDA topic modeling, a method of natural language processing, to draw the motivating factors of urban forest visitors. The unsupervised problem method was used to identify the factors within a vast scope of data. In the context of LDA modeling, generally boxes are referred as “plates” which represents replicates. In other words, replicates represent documents in outer plate. Inner plate represents the repeated choice of topics and words within a document, where the marginal distribution of a document is obtained by the repeated choices of the converted document-term matrix [31]. Finally, we obtained the probability of a corpus through aforementioned statistical analysis, and presented the scores of the words in topics with the highest probability of correlation. Using this method, the scores of keywords are listed in order from the highest probability of correlation with a given topic, based on the keywords within the topic, as detected in the LDA analysis. The potential keywords for each topic were thus obtained. A schematic of the LDA topic modeling algorithm is shown in Figure 1.

LDA topic modeling automatically extracts specific topics, issues, or theme groups that represent a given set of texts, with a focus on the pattern of concurrently used keywords present in the text data. Here, a topic may be viewed as a group of words sharing similar senses with a high probability of being detected together. The method allows the extraction of potential topics (factors) based on the statistical analysis of the frequency of words within the text data enabling frame analysis, which is useful for relatively quickly understanding the online community and inferring possible trends within the community [32]. Notably, the LDA method, compared to the other topic modeling techniques, is advantageous in drawing a number of different topics (frames) from a vast scope of informal data, as it leads to ready interpretation of results and solves the problem of overfitting.

Such an algorithm is especially useful for identifying hidden topics and themes based on the word co-occurrence for each document in the corpus. It can be applied when the task requires the analysis of topics across a large number of documents since it is relatively simple for a person to analyze the topics in one or two documents, but the analysis of topics across hundreds or thousands of different documents is not easy. In addition, topic modeling is a good way to “let the text talk” because the identified topics do not depend on the evaluators’ individual perspectives or experiences. Moreover, there are numerous possible extensions of LDA, hence LDA topic modeling has been recently applied across fields [31,33,34]. In Figure 1, the hyperparameter is the topic number set directly by the user. It is the number that determines the specific number of words, that is, the number of theme words and keywords contributing to the topic, while the optimum number of documents depends on the number of topics determining the number of clusters that point toward the theme words. The formation of a single topic is thus proceeded by converging the words with a high probability contribution, and if the number of words under a topic is too high or low, the interpretation of the deduced topic will be difficult. If the number of topics is too high or low, the words within each topic may be duplicated to be eliminated or the topic may show a reduced correlation to pose challenges in interpretation. Many researchers who utilize topic-modeling methods point out that loss of interpretability is a major limitation of complex learning algorithms such as topic models, that is to say, it is important to note that topics produced by complex algorithms are numeric values based on mathematical properties, but the interpretation of them depends on the goals of the analysis, the researcher’s perspectives and domain knowledge [35,36,37,38]. Thus, the hyperparameter, as directly set by the user, should allow the optimum number to be determined through several reasonable attempts based on the experimental values or scores for the topics to be deduced. In addition, either by asking an expert to annotate the topics or by conducting a repeated LDA process with an expert’s consultation, topic keywords are found for each data set. Additionally, in order to make it easier to distinguish primary attributes of the derived topics, researchers qualitatively designated each name of the topic group [39,40]. With this process, the topic names, that is, theme words, are defined by the investigator based on the words contributing to each topic obtained through LDA topic modeling for the reinterpretation of the results.

## 4. Results

### 4.1. Motivating Factors of Urban Forest Visitors (LDA Topic Modeling) 

The topics (factors) were drawn from the keywords belonging to the set clusters through discussions between the present investigator and the authors of previous studies. Specifically, based on the words showing high frequency, the words with high correlation were analyzed for probability, and each topic cluster was defined according to the probability distribution (cosine coefficient), which was then named as the theme word to deduce a total of seven topics. As a conventional procedure in LDA-based topic identification, each topic was manually named after analyzing the words included in topics [41]. To ensure the identification of topics with high relevance to motivating factors of urban forest visitors, the topics and words in Table 2 were compared with the items of motivation that have been often examined in leisure and outdoor recreation motivation-related studies, especially mentioned in Table 1. Based on the in-depth comparison, the authors initially generated the name of topics that best describes words included in each topic. The names of the topic were finally confirmed based on the agreements from all authors. The results of the LDA topic modeling for the motivating factors of urban forest visitors are shown in Table 2.

Words with a high vector-to-vector correlation were grouped according to the score. Based on the result and throughout the agreement among researchers, the topics of the group were named. Thus, the seven identified topics (factors) were “Cafe-related Walk”, “Healing Trip”, “Daily Leisure”, “Family Trip”, “Wonderful View”, “Clean Space”, and “Exhibition and Photography”. 

### 4.2. Interpretation for the Motivating Factors

The first motivating factor, “Cafe-related Walk” contained the keywords “cafe, bicycle road, cottage, coffee, road, trip, and night.” Based on the keyword “cafe”, the connection may be made to “bicycle road”, “coffee”, and “trip”, which can indicate the coffee enjoyed at a cafe, a trip on a bicycle, and a walk along the road. Koo et al.’s [42] survey of visitors to the Seoul Forest in South Korea showed that exercise and rest were the two most frequently detected motivating factors. Based on this, it may be interpreted that the cafe and bicycle road acted as critical external factors. In other words, visitors to urban forests may be interpreted as enjoying a night walk or bicycle ride to the forest to enjoy coffee.

The second motivating factor, “Healing Trip” contained the keywords “trip, summer, travel site, healing, scenery, nature, and photography”. Based on these keywords, visitors to urban forests may be interpreted as visiting the travel site mainly during summer to enjoy the natural scenery and take photographs, and as indicated by the word “healing” that corresponds to an internal motivation for rest, health, and personal desire, the visitors showed a strong correlation with leisure, such as a healing trip, whereby they could photograph the beautiful natural scenery.

The third motivating factor, “Daily Leisure” contained the keywords “cafe, street, life, home, location, walk, and vicinity”. The keyword “cafe” here indicated a different meaning from the one contained in “Cafe-related Walk”, and while it exhibited the highest score, the word may be interpreted to describe a daily life in an urban area. As the meaning was related to walking along the streets surrounding the residential area, the theme was closer to daily leisure than to trip. Visitors can be interpreted as visiting the forest as part of daily leisure and spending time in the vicinity of cafes close to home.

The fourth motivating factor, “Family Trip” contained the keywords “gourmet, tourism, trip, location, sea, family, and course”. Based on the keyword “family”, the main motivating factors can be interpreted as the visit to gourmet restaurants, tourism, and trip. The theme is closer to trip than daily leisure, and visitors can be seen to visit the forest to enjoy a gourmet restaurant nearby or as part of tourism or trip.

The fifth motivating factor, “Wonderful View” contained the keywords “famous site, city, autumn, maple, dream, tree, environment”. It is characterized by the goal of visiting the famous forest in the suburban area, enjoying the beautiful scenery of the season. The keywords “autumn”, “maple”, and “tree”, in particular, indicated the scenery of a beautiful forest as a famous site in autumn. This result is supported by a previous study [10] showing the correlation of a beautiful scenery with the satisfaction of urban forest visitors.

The sixth motivating factor, “Clean Space”, contained the keywords “fine dust, itinerary, park, citizen, tree, river, sea”. A notable keyword among them is “fine dust” that may indicate the keywords “park”, “tree”, “river”, and “sea” as external factors of motivation to be away from the city. The number of tourists leaving in search for a natural site known to have refreshing air is on the rise, and with increased interest in rest and healthcare, the goal of visiting an urban forest may be interpreted as the desire to visit a clean space.

The seventh motivating factor, “Exhibition and Photography”, contained the keywords “exhibition, photography, gourmet, popularity, display, winter, and nature”. The exhibitions related to forests and the goal of photography motivated urban forest visitors. In fact, an increasing trend of visit was found for exhibition galleries, museums, and experience centers surrounding urban forests, and as events such as urban forest cultural experience are being held, the goal of the visitors may be interpreted as the photographing of a popular gourmet restaurant or famous site, and the theme word was named as “Exhibition and Photography”.

## 5. Discussion

To investigate the motivating factors of urban forests visitors, this study used social big data from Naver and Daum blogs with 60% share of portal sites in South Korea (based on 2020). A total of 57,449 cases of data from 2019 to 2020 were collected from the blogs related to urban forest visitors; after data cleaning, the final 40,110 cases of data were used to extract the motivating factors of urban forest visitors through text mining and LDA topic modeling techniques. Based on these results, pedagogical and practical implications were drawn.

First, to identify the motivating factors of urban forest visitors, no factor was predetermined, and the factors were extracted from the data of visitor experience on the websites that were collected and analyzed comprehensively. In this way, methodological efforts were made to minimize the subjective judgment frequently observed in conventional scale-development methods that use literature review and in-depth interview to extract initial factors and in turn, conduct survey and exploratory or confirmatory factor analysis to finalize major factors.

Second, in previous studies applying conventional factor analysis, the small sample size posed limitations due to time and cost constraints; in this study, the use of continuously accumulated social data allowed the collection of a large body of samples in a short period, and the limitation of spatial range was possibly transiently overcome. For this, the target sites were not urban forests, forestry, mountains, and green areas in a specific region, but by using social big data and the keyword “urban forest”, a more extensive scope of regions could be analyzed.

Third, while limitations were found in previous studies based on a cross-sectional design and with a focus on a transient time, the LDA topic modeling in this study allowed the incorporation of temporal variations that reflect a wide scope of time to provide the data through which the motivating factors could be analyzed based on the visitors’ experiences. Urban forest visitors in modern society are influenced by online media, but only a few studies have used social data. Thus, the academic significance of this study lies in suggesting a novel method of analysis based on social media data. In addition, using LDA topic modeling, this study contributed to introducing more concrete motivating factors of urban forest visitors. Unlike other forest-related studies, this study specially presented the motivating factors related to tourism and leisure activities in detail such as ‘café-related walk’, ‘family trip’, etc. Based on the findings, the study was able to propose diverse insights and perspectives for designing and managing urban forests.

An overall interpretation of the results for the motivating factors of urban forest visitors showed that due to the recent phenomenon of fine dust, the preference of visitors towards clean space has increased, while most urban forest visitors walk around cafes or enjoy the scenery. The themes of “Healing” and “Photography” as daily leisure or time in nature (forest) were shown to be preferred by visitors to urban forests. Based on the motivating factors identified from social big data, the following practical implications are suggested.

First, among the motivating factors of urban forest visitors, “Cafe-related Walk” and “Daily Leisure” were both found to be based on the keyword “cafe”. The results implied that many tourists visited an urban forest to visit a nearby café. The results showed that their motivations to visit urban forests were to take a trip on a bicycle, enjoy daily leisure activities such as walking, and enjoy the leisure of drinking coffee at a cafe. Thus, accessibility to urban forests may be improved through the development of tourism packages connecting urban forests and cafes and the construction of roads such as bicycle roads around urban forests, as urban forest visitors preferred daily leisure.

Second, “Healing Trip” and “Clean Space” clearly indicated the increased number of people seeking the healing of the body and mind and the clean space without fine dust, in line with the recent increase in the desires for rest and health due to the COVID-19 pandemic and the find dust phenomenon. As such, marketing managers should make efforts to highlight the benefits of urban forests as a clean space without finding dust and a place for the slow healing of the body and mind, while visitors may take the rest in nature and photograph the beautiful scenery.

Third, “Family Trip” and “Wonderful View” showed that, for urban forest visitors, the tour of gourmet restaurants and wonderful view were critical motivating factors. The urban forests known as famous sites were visited for the beauty of a specific season as indicated by the keywords “autumn”, “maple”, and “tree”, while the goals of the family in visiting urban forests were the tour of gourmet restaurants and trip. Thus, to increase the number of visitors to urban forests, ways should be sought to introduce accommodations for family visitors together and promote famous and beautiful gourmet restaurants and historic sites.

Fourth, “exhibition” and” photography” were found among the motivating factors to indicate the importance of exhibitions and photography related to urban forests. The exhibition galleries, museums, and experience centers related to urban forests as well as events such as urban forest cultural experience contributed to these themes as important motivating factors. The Green Walking Project in the UK contains photography, meditation, and forest experience as part of a program with flexibility for modification in accordance with the circumstances in the field [43]. In reference to this, efforts should be made to continuously expand projects related to exhibitions and experience centers in urban forests and to offer various activities and educational programs for exhibition and photography.

Last but not least, the benefit of urban forests needs to be mentioned. The results of this study showed that urban forests can play important roles in not only improving urban dwellers’ public health such as physical and mental health but also increasing leisure and recreational activities. In particular, the study revealed that urban forests can serve as a tourism or leisure destination in the city beyond simply providing a green space for rest or healing. Looking at the collected text data, many visitors frequently used the word ‘travel’ and ‘trip’ concerning visiting urban forests. This might mean that not only urban residents living close to urban forests but people living farther than expected can benefit from urban forests. With regards to the health impact of urban forests, as Gianfredi et al.’s [44] recent study about urban greenspace and health found, there are positive associations between urban greenspace exposure and the level of physical activity and mental health outcomes such as well-being, ease of stress, and quality of life. This result stresses that although this study showed the important aspects of urban forests as a space for daily leisure and tourism, the urban forests should maintain the basic functions and benefits for improving and facilitating public health.

With regards to the limitation of this study, the motivating factors based on the characteristics of urban forests in each region or administrative district could not be analyzed as the factors were extracted from the social big data-based web data that had been comprehensively collected. Nevertheless, the general factors that motivate the visit to urban forests across the nation can be identified extensively. For example, the urban forests within the city and in a suburban area may vary in terms of the location and travel range to affect motivation, and as each region or administrative district has different characteristics, it may be difficult to generalize the results of this study. More focused and specific studies should be conducted to determine the general motivating factors of urban forest visitors and to suggest a paradigm through which conventional methods are complemented in various ways. Further studies should also continue to investigate the differences between the forests within the city and in a suburban area and the regional characteristics or the unique features of each urban forest to determine the various motivating factors, which will lead to greater academic significance.

The information analyzed might have a sample bias because all the samples collected were visitors who are encouraged to post their experiences online after visiting urban forests. This means that those visitors who are less active to leave their comments on blogs might have been neglected, which in turn, results that the motivating factors extracted may not be fully representative of all urban forest visitors. To reduce the bias, it is suggested to use the mixed method that incorporates both text mining and in-depth interview in future studies. In addition, the study was conducted only in one geographical context and, balanced sampling considering demographic distribution such as age, gender, educational level, etc., was not performed. This may mean that the findings and implications may not fully apply to other geographical settings. Therefore, it is suggested to conduct cross-region or cross-cultural studies to validate the results of this study and to provide more insightful explanations about the cultural and geographical impact on motivations.

## Figures and Tables

**Table 1:** The factors that motivate the visit to urban forests.

Category		Motivating Factors	Previous Studies
Forestry	urban forest (park)	privacy benefits of urban forest visitors (privacy protection)	Hammitt [16]
		desire for rest, social interaction, sense of belonging, technical prowess and knowledge	Pan and Ryan [9]
		physical exercise, rest, interaction with nature, visiting with a child, enjoying good weather, visiting cultural heritage sites, clean and fresh air, reading books, meeting friends, walking dogs	Liu, Li, Xu and Han [17]
		Socially oriented motivation, nature-oriented motivation	Zhai, Baran and Wu [18]
	healing forest	physical motivation, psychological motivation, social motivation, natural environment, ancillary facilities, programs and field experiences	Nam and Lee [8]
		natural experience, calmness, natural exploration, health, social interaction, emotional relaxation	Kim and Kim [7]
	natural recreation forest	enjoying the natural environment, escape from daily life, new trip, healthy life, sense of intimacy	Kim, Lee, Uysal, Kim and Ahn [19]
		education of a child, escape from daily life, novelty, natural exploration, social interaction, use of programs or facilities, amenities, ecology or environment	Choi and Lim [6]
	urban green space	forest walk, social activity, bathing and cycling, rest, picnic, food tour, peacefulness, botanical study, festival, study of people and heritage in cities, animal or dog-related activity, rides, ball games, sports, arts, toys	Stigsdotter and Grahn [20]
		enhanced health, facilitated child development, reduced stress, increased financial values, contact with nature, importance of daily life, space of social interaction	Jim and Shan [13]
national park		rest or hobby, novelty (new activity), reinforced social relationship, escape from daily life, reputation	Uysal, McDonald, and Martin [21]
		studying the nature, relieving stress or solitude, pursuing a challenge, adventure or sense of accomplishment, self-perception, career position, sense of belonging, fun, autonomy	Confer, Vogelsong, Graefe, and Solan [22]

**Table 2:** Result for LDA Topic-Modeling of Urban Forest.

Topic	Words	Score	Topic	Words	Score
Cafe-related Walk	Cafe	0.016	Healing Trip	Trip	0.012
	Bicycle road	0.011		Summer	0.006
	Cottage	0.007		Travel site	0.006
	Coffee	0.007		Healing	0.005
	Road	0.006		Scenery	0.005
	Trip	0.005		Nature	0.005
	Night	0.005		Photography	0.004
Daily Leisure	Cafe	0.027	Family Trip	Gourmet	0.035
	Street	0.006		Tourism	0.007
	Life	0.006		Trip	0.006
	Home	0.004		Location	0.006
	Location	0.004		Sea	0.005
	Walk	0.004		Family	0.005
	Vicinity	0.004		Course	0.005
Clean Space	Fine dust	0.008	Wonderful View	Famous site	0.006
	Itinerary	0.006		City	0.006
	Park	0.004		Autumn	0.004
	Citizen	0.003		Maple	0.004
	Tree	0.003		Dream	0.003
	River	0.003		Tree	0.003
	Sea	0.003		Environment	0.003
Exhibition and Photography	Exhibition	0.01			
	Photography	0.009			
	Gourmet	0.009			
	Popularity	0.008			
	Display	0.006			
	Winter	0.006			
	Nature	0.006			

## Data Availability

Not applicable.
